# Exenatide Activates the APPL1-AMPK-PPAR*α* Axis to Prevent Diabetic Cardiomyocyte Apoptosis

**DOI:** 10.1155/2016/4219735

**Published:** 2015-11-30

**Authors:** Lei XiaoTian, Wu QiNan, Gan XiaGuang, Deng WuQuan, Chen Bing, Liang ZiWen

**Affiliations:** Endocrine Department, The First Affiliated Hospital of the Third Military Medical University, Chongqing 400038, China

## Abstract

*Objective*. To investigate the effect and mechanism of the exenatide on diabetic cardiomyopathy. *Methods*. Rats were divided into control group, diabetes group (D), diabetes treated with insulin (DI) group, and diabetes treat with exenatide (DE) group. We detected apoptosis rate by TUNEL, the adiponectin and high molecular weight adiponectin (HMW-adiponectin) by ELISA, and the expression of APPL1, p-AMPK/T-AMPK, PPAR*α*, and NF-*κ*B by immunohistochemistry and western blotting. *Results*. Compared with the D group, the apoptosis in the Control and DE groups was decreased (*P* < 0.05); the adiponectin and HMW-adiponectin were increased (*P* < 0.05); the APPL1, p-AMPK/T-AMPK, PPAR*α*, and LV −*dP/dt* were increased (*P* < 0.05); and the NF-*κ*B, GRP78, and LVEDP were decreased (*P* < 0.05). Compared with DE group, the glucose levels in the DI group were similar (*P* < 0.05); the apoptosis and LVEDP were increased; the APPL1, p-AMPK/T-AMPK, PPAR*α*, and LV −*dP/dt* were decreased (*P* < 0.05); the NF-*κ*B and GRP78 were increased (*P* < 0.05); the adiponectin and HMW-adiponectin were significantly decreased (*P* < 0.05). *Conclusion*. Our model of diabetic cardiomyopathy was constructed successfully. After being treated with exenatide, the adiponectin and HMW-adiponectin and the APPL1-AMPK-PPAR*α* axis were increased, the NF-*κ*B and the apoptosis were decreased, the cardiac function of the diabetic rats was improved, and these effects were independent of glucose control.

## 1. Introduction 

Diabetic cardiomyopathy is common in diabetic patients. Many researchers have found that cardiomyocyte apoptosis plays an important role in diabetic cardiomyopathy [1–3]. Therefore, inhibiting cardiomyocyte apoptosis is the key step to prevent diabetic cardiomyopathy. Adiponectin is one of the highest concentration adipokines in blood serum, and its concentration is associated with diabetic cardiovascular complications and other cardiac diseases. Adiponectin has also been suggested to be a risk factor for such complications [4]. Previous researchers have indicated that adiponectin can protect cardiomyocytes by activating APPL1, a protein that interacts with adiponectin and its receptor to stimulate AMPK and PPAR*α* and plays a central role in the adiponectin signaling pathway in ischemia-reperfusion models. However, the mechanism of APPL1 activity is unclear, and there is little research about the role of APPL1 in diabetic cardiomyopathy and cardiomyocyte apoptosis [5, 6]. Recent reports have suggested that glucagon-like peptide-1 (GLP-1) agonists and DPP-4 inhibitors can stimulate the secretion of adiponectin and that this effect is independent of glucose reduction [7]. Whether the adiponectin signaling pathway is the main mechanism by which GLP-1 reduces cardiomyocyte apoptosis is still unclear. We constructed a type 2 diabetes rat model by streptozotocin (STZ) injection and a high-fat diet to investigate the effect and mechanism of exenatide, a GLP-1 receptor agonist, on diabetic cardiomyopathy and cardiomyocyte apoptosis.

## 2. Material and Methods

### 2.1. Animals Preparations

A total of 40 healthy 4-week-old male Sprague Dawley (SD) rats weighing 120–150 g were obtained from the animal center of the Third Military Medical University, Chongqing. The rats were placed in a room with controlled lighting (12-hour light/dark cycle) and regulated temperature (18–25°C) and humidity. All of the rats were fed with regular chow and water for 7 days to allow them to adapt to the environment. After 7 days, eight rats were randomly selected as the normal Control group (control), and they continued to eat a normal chow diet. The remaining rats were given STZ at a dose of 35 mg/kg (SIGMA company, dissolved in citrate buffer, pH 4.5) via intraperitoneal injection and fed a high-fat diet (HFD, which contains 33% carbohydrate, 13% protein, and 54% fat) for 72 hours. After 72 hours of STZ injection and HFD feeding, we obtained blood samples from the rats' tail veins to test the blood glucose levels (Abbott Laboratories' glucometer and test paper). A random blood glucose level >16.7 mmol/L in the rats was considered diagnostic of type 2 diabetes [8]. We randomly selected 24 rats that had reached this standard and divided them into 3 groups. The D group was fed a HFD and received daily saline injections for 12 weeks (*n* = 8); the DI group was fed a HFD and received daily subcutaneous injections of premixed insulin 30R (Novolin R; Novo Nordisk, Copenhagen, Denmark) at a dose of 0.4 U/kg to control their hyperglycemia for 12 weeks (*n* = 8); and the DE group was fed a HFD and received daily subcutaneous injection of exenatide (Amylin Corporation, San Diego, CA, USA) at a dose of 0.5 *μ*g for 12 weeks [9]. The animals' weights and blood glucose levels were measured daily.

### 2.2. Cardiac Function and Heart Morphology

The rats were anesthetized using 10% chloral hydrate. Their cardiac function and heart morphology were evaluated at multiple time points using echocardiography (Vevo 2100, VisualSonics). The left ventricular systolic pressure (LVSP), left ventricular end diastolic pressure (LVEDP), and mean arterial pressure (MABP) were detected as described previously [10].

### 2.3. Immunohistochemistry

The rats were anesthetized using 10% chloral hydrate, and cardiac tissues were collected for examination. Immunohistochemical analysis of paraffin-embedded tissue was performed on the hearts of all rats. Five-micrometer thick paraffin sections were deparaffinized and rehydrated. For antigen retrieval, the sections were microwaved in distilled water for 10 min followed by washing in phosphate-buffered saline (PBS) for 5 min. The deparaffinized sections were then incubated with primary antibodies as follows: APPL1 (dilution 1 : 300; Santa Cruz, USA), p-AMPK and T-AMPK (dilution 1 : 200; Santa Cruz, USA), PPAR*α* (dilution 1 : 400; Santa Cruz, USA), and NF-*κ*B (dilution 1 : 200; Santa Cruz, USA) overnight at 4°C. The sections were washed with PBS and then incubated with horseradish peroxidase- (HRP-) labeled goat antirabbit antibody (dilution 1 : 100; DAKO, Glostrup, Denmark) for 30–60 min at room temperature. The sections were then washed three times in PBS and incubated at room temperature with 3,3′-diaminobenzidine (DAB) (DAKO, Glostrup, Denmark).

### 2.4. Western Blotting

After lysis buffer was added to cardiac tissue, total protein was extracted, and the concentration was measured. Equal amounts of protein preparations were separated by sodium dodecyl sulfate-polyacrylamide gel electrophoresis (SDS-PAGE) for 30 min at 80 V; then, the separated proteins were transferred to nitrocellulose membranes (Boer Biotechnology Company) for 60 min at 120 V. The membranes were blocked with 5% nonfat milk (SIGMA, USA) in PBS with 0.05% Tween-20 (PBST, pH 7.6) for 2 h and then incubated with the following primary antibodies: APPL1 (1 : 500, Santa Cruz), p-AMPK and T-AMPK (1 : 1000, Santa Cruz, USA), PPAR*α* (1 : 500 and 1 : 1000, Santa Cruz, USA), and NF-*κ*B (P65) (1 : 600, Santa Cruz, USA) at 4°C overnight. The membranes were washed with TBST and then incubated with 1 : 5000 HRP-conjugated antirabbit IgG (Santa Cruz, USA) for 90 min on a tabletop incubator at 50 rpm and 37°C. The membranes were then washed again with TBST. The membranes were scanned with Typhoon (Pharmacia, USA) and quantitated using Quality One. We detected the protein expression level 3 times for each sample.

### 2.5. Apoptosis Detection

Cardiac tissues were rinsed 3 times in PBS and then fixed in 4% paraformaldehyde. The samples were then digested with proteinase K and incubated with TUNEL liquid for 90 min at 37°C. The reaction was terminated by adding a sodium citrate solution. The samples were then incubated with anti-HRP antibody for 15 min, washed with PBS, and stained with DAB. The samples were observed and photographed at 400x on a light microscope, and the apoptosis rate was calculated as previously described [11].

### 2.6. Statistics Analysis

The data are shown as the mean ± standard deviation (*X* ± *S*) and were analyzed using SPSS 19.0. The nonparametric rank sum test and analysis of variance (ANOVA) were used, and *P* < 0.05 indicated a significant difference.

## 3. Results

### 3.1. Modeling and the Effects of Type 2 Diabetes in Rats

Compared with the levels in the Control group, the body weight of the D group was significantly increased (from 426 to 491 g, *P* < 0.05), as were the plasma concentrations of triglyceride (TG) and total cholesterol (TC). The fasting blood glucose (FBG) of the D group was significantly increased compared with that of the Control and other groups. Additionally, the HOMA-IR, which is an index of insulin resistance, was significantly increased in the D group compared with the Control group. These findings indicated that the type 2 diabetes rat model was constructed successfully. The plasma glucose concentrations did not significantly differ between the DI and DE groups, whereas the plasma insulin levels tended to be increased in the DI group. Compared with the Control and D groups, the DE group showed both significantly reduced body weight, HOMA-IR, and the TG and TC levels and increased concentrations of adiponectin and HMW adiponectin after exenatide (0.5 *μ*g/d) treatment (Table 1).

### 3.2. Cardiac Function in Each Group

**Figure 1:**
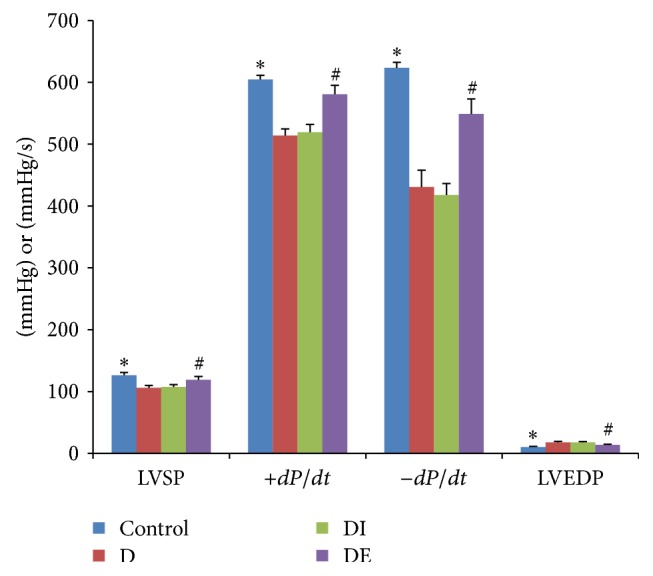
The cardiac function of each group. *∗* compared with the D group, *P* < 0.05; # compared with the DI group, *P* < 0.05.

We analyzed the cardiac function results by using a multimedia biology signal recorder (Figure 1 and Table 2). Compared with the Control group, the D group showed markedly reduced LV +*dP/dt*, while the LVEDP was increased significantly. We observed that the cardiac function of diabetic rat was impaired compared with normal rats. Compared with the D group, the DE group showed markedly increased LV +*dp/dt*, while the LVEDP was markedly reduced; all of the differences were statistically significant (*P* < 0.05). However, these parameters did not differ between the D and DI groups. The glucose level was similar between the DE and DI groups. However, for the rats in the DI group, the LV +*dP/dt* was markedly reduced, and the LVEDP was markedly increased compared with the DE group; all of these differences were statistically significant (*P* < 0.05). To summarize, these findings suggest that our model of diabetic cardiomyopathy was constructed successfully and that exenatide could effectively ameliorate the impaired cardiac function compared with insulin treatment. In addition, the therapeutic effect was independent of glucose control.

### 3.3. Cardiomyocyte Apoptosis in Each Group

**Figure 2:**
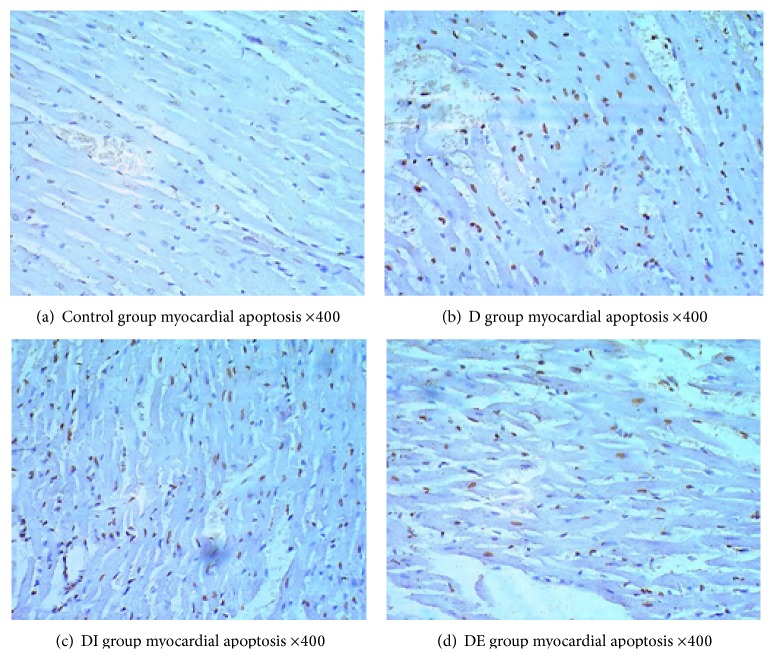
Myocardial apoptosis in each group ×400.

**Figure 3:**
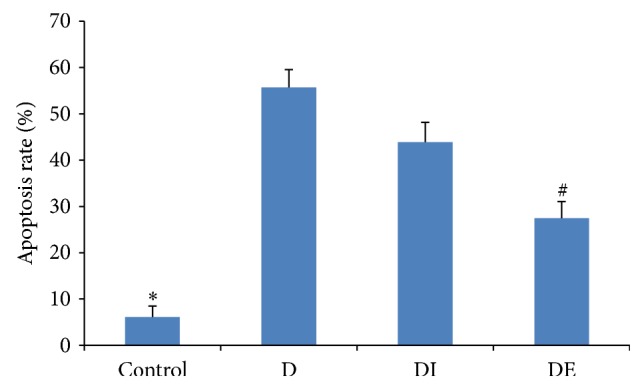
The apoptosis rate in each group. *∗* compared with the D group, *P* < 0.05; # compared with the DI group, *P* < 0.05.

Interestingly, the apoptosis rate of the DE group was significantly decreased compared with that of the D and DI groups (*P* < 0.05). Compared with the apoptosis rate in the Control group, the apoptosis rates in the D group and DI group were markedly increased (*P* < 0.05) (Table 3, Figures 2 and 3). These findings demonstrated that exenatide could decrease the rate of diabetic cardiomyocyte apoptosis and improve cardiac function.

### 3.4. The Expression of APPL1, p-AMPK/T-AMPK, PPAR*α*, NF-*κ*B, and GRP78 in Each Group

**Figure 4:**
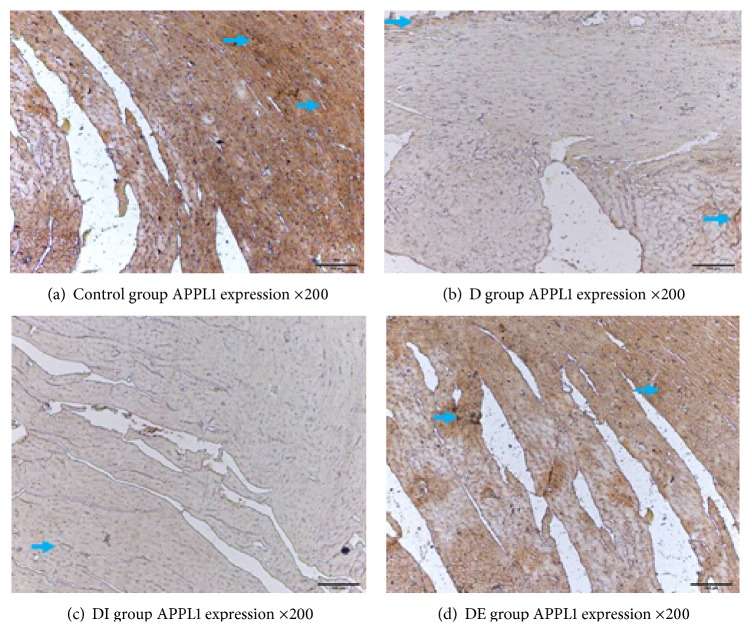
APPL1 expression in each group ×200.

**Figure 5 fig5:**
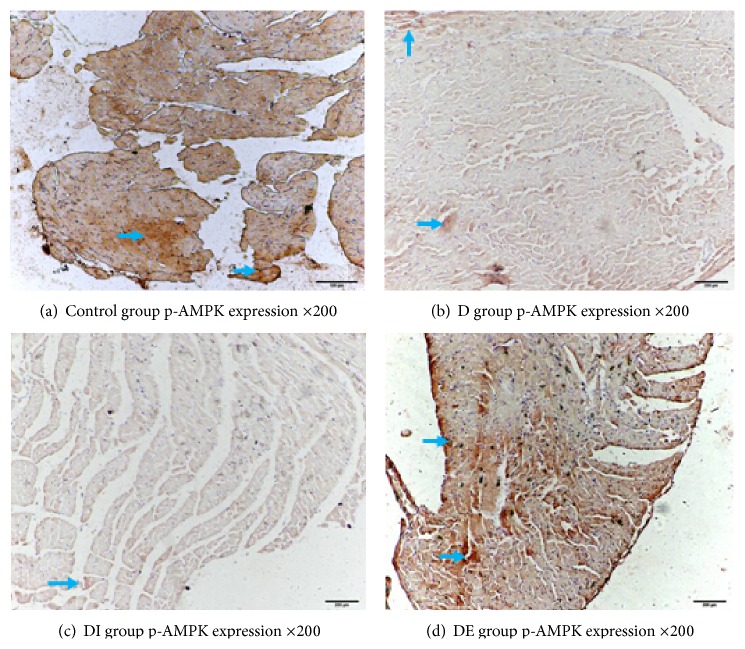
p-AMPK expression in each group ×200.

**Figure 6 fig6:**
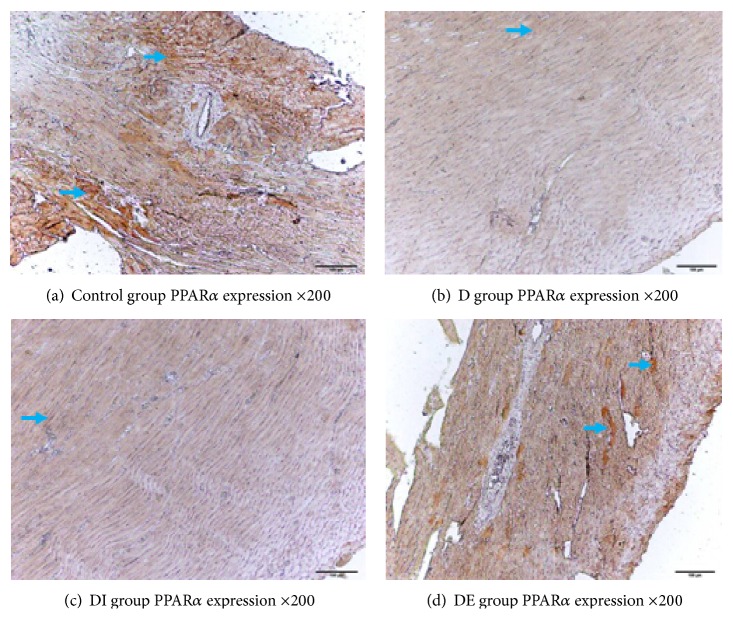
PPAR*α* expression in each group ×200.

**Figure 7:**
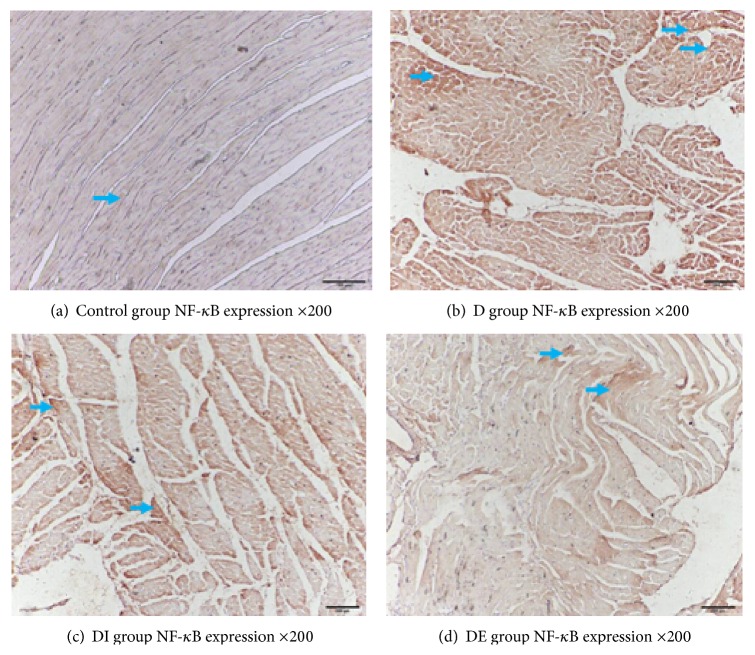
NF-*κ*B expression in each group ×200.

We detected the cardiac expression of APPL1, p-AMPK/T-AMPK, and PPAR*α* by immunohistochemistry. Staining for APPL1, p-AMPK, and PPAR*α* in the DE group was increased compared with the D and DI groups, but the NF-*κ*B level in the DE group was lower compared with the D and DI groups. These findings demonstrate that APPL1, p-AMPK/T-AMPK, and PPAR*α* expression was lower while NF-*κ*B had higher expression in type 2 diabetic rats. After exenatide treatment, the levels of APPL1, p-AMPK/T-AMPK, and PPAR*α* expression were decreased, and the expression of NF-*κ*B was increased; insulin treatment had no effect on the expression of APPL1, p-AMPK/T-AMPK, and PPAR*α* (Figures 4 [Fig fig5] [Fig fig6]–7).

**Figure 8:**
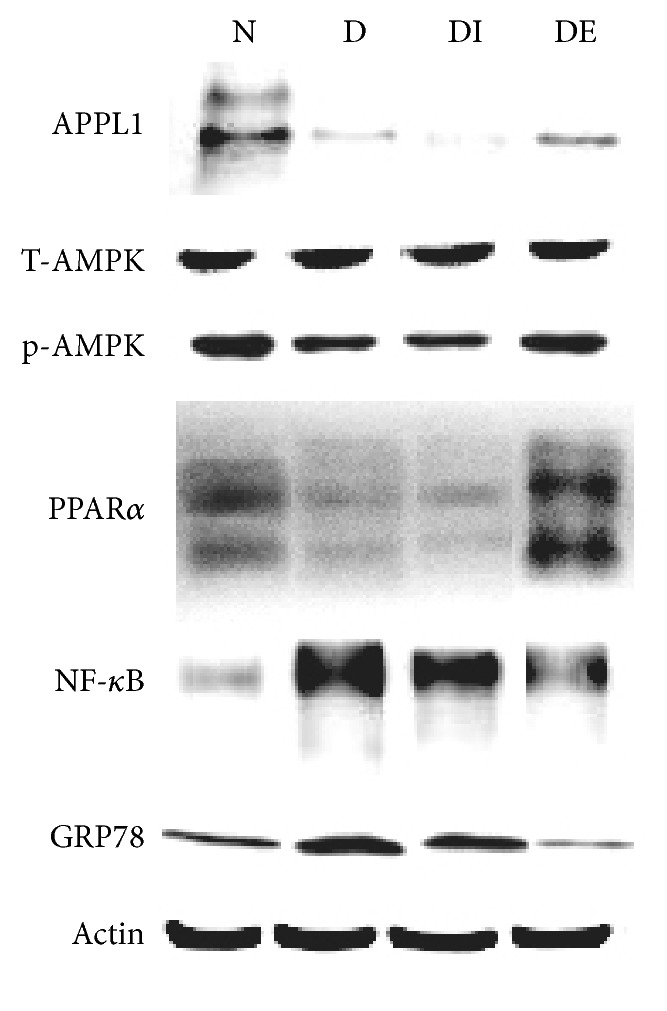
Protein expression levels of APPL1, p-AMPK/T-AMPK, PPAR*α*, GRP78, and NF-*κ*B in each group.

**Figure 9:**
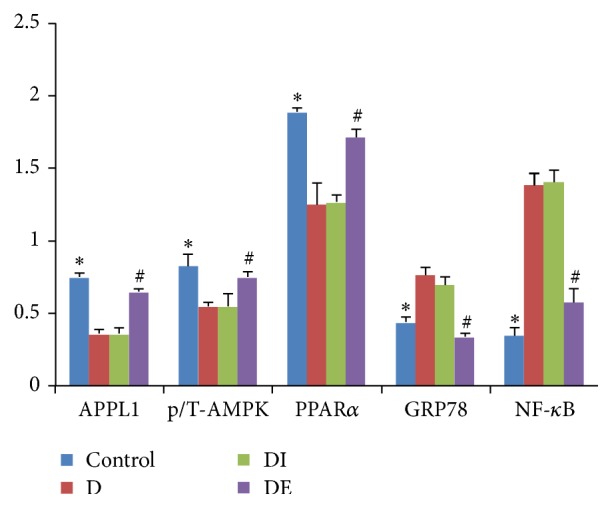
The protein expression levels of APPL1, p-AMPK/T-AMPK, PPAR*α*, NF-*κ*B, and GRP78 in each group. *∗* compared with the D group, *P* < 0.05; # compared with the DI group, *P* < 0.05.

The expression of APPL1 in the DE group was increased compared with that of the D and DI groups. The expression of APPL1 in the D group was significantly decreased compared with that of the Control group (Figures 8 and 9, Table 4, *P* < 0.05).

The expression of p-AMPK/T-AMPK in the DE group was increased compared with that of the D and DI groups. The p-AMPK/T-AMPK level in the D group was significantly decreased compared with the Control group (Figures 8 and 9, Table 4, *P* < 0.05).

The expression of PPAR*α* in the DE group was increased compared with that of the D and DI groups. The PPAR*α* expression in the D group was significantly decreased compared with the Control group (Figures 8 and 9, Table 4, *P* < 0.05).

Interestingly, in the DE group, the expression of NF-*κ*B and GRP78 was lower than that in the D and DI groups. The expression of NF-*κ*B and GRP78 in the D group was significantly higher than in the Control group (Figures 8 and 9, Table 4, *P* < 0.05).

## 4. Discussion

Type 2 diabetes and its cardiovascular complications are leading causes of mortality and morbidity among diabetic patients in China. Recently, researchers have found that diabetic cardiovascular complications have a common end outcome: diabetic heart dysfunction. A large number of reports have revealed that cardiomyocyte apoptosis plays an important role during the pathogenesis of diabetic cardiovascular complications [1, 2]. Long exposure times to metabolic perturbations can induce ER stress and initiate the apoptosis signaling pathway, leading to cardiomyocyte apoptosis, diabetic cardiomyopathy, and cardiac dysfunction and failure [2]. However, the mechanism of these outcomes is unclear.

GLP-1 is a hormone that is secreted by intestinal L cells, and GLP-1 can promote the secretion of insulin and decrease the secretion of glucagon. Additionally, recent research suggests that GLP-1 can protect against cardiovascular disease. In rats with myocardial ischemia reperfusion injury, GLP-1 treatment can reduce cardiomyocyte apoptosis and infarct size [12]. Other studies have also revealed that this protective effect of GLP-1 is independent of the serum glucose concentration [7], but the protective mechanism is unclear.

Recently, many reports have suggested that GLP-1 agonists and DPP-4 inhibitors can promote adiponectin secretion [13–15]. Adiponectin is the most concentrated adipocyte factor in plasma and is also a cardiovascular protection factor. Adiponectin binds to its receptor AdipoR1/2 and APPL1 and then activates AMP-activated protein kinase (AMPK), reducing oxidative stress, and inhibits NF-*κ*B, preventing endothelial cell apoptosis and dysfunction [2, 3]. Park et al. reported that palmitic acid can induce ER stress, decreasing the adiponectin effect and inducing cardiomyocyte apoptosis [16]. However, this phenomenon has not been reported in diabetic cardiomyocyte apoptosis [6, 7]. Thus, it is still unclear whether the mechanism of GLP-1 agonists to decrease cardiomyocyte apoptosis in diabetes is associated with adiponectin.

PPAR*α* is a key protein and a downstream target of AMPK in the adiponectin signaling pathway [17]. Some researchers have discovered an interesting relationship between GLP-1 receptor and PPAR*α*. Maida et al. demonstrated that metformin increases the expression of the GLP-1 receptor in pancreatic beta cells, and this increase was attributed to the action of PPAR*α* because no incretin receptor gene expression increase was observed in PPAR*α*-knockout mice in vivo. This finding suggested that metformin can directly increase GLP-1R expression via a PPAR-*α*-dependent mechanism [18]. Additionally, high doses of DPP-4 inhibitor can increase PPAR*α* expression in brown fat, and the mechanism is at least partially related to GLP-1 [19]. Another study has shown that GLP-1 also has a direct effect on hepatocytes by activating genes involved in PPAR*α*-related fatty acid *β*-oxidation and insulin sensitivity [20]. Many researchers have reported that PPAR agonists may represent a new approach for managing type 2 diabetes via modification of endogenous GLP-1 secretion [21]. However, whether this mechanism plays a role in diabetic cardiomyopathy is unclear. In previous studies, we found that PPAR*α* could inhibit NF-*κ*B (a protein complex that is involved in cellular responses to stimuli such as stress, cytokines, free radicals, ultraviolet irradiation, oxidized LDL and chronic inflammation). NF-*κ*B plays a key role in regulating the immune response to chronic inflammatory conditions such as diabetes, and it can directly interact with PPAR*α* to inhibit fatty acid oxidation and oxidative stress, protecting cardiomyocytes from apoptosis [11]. However, the upstream signaling pathway has not been elucidated. Whether GLP-1 agonists promote APPL1-AMPK-PPAR*α* signaling to decrease NF-*κ*B expression and cardiomyocyte apoptosis in diabetes is still unknown [22, 23].

In our research, the diabetic rats had significantly higher TC and TG concentrations, NF-*κ*B expression, apoptosis rates, and LVEDP (*P* < 0.05) but significantly lower levels of adiponectin and HMW adiponectin: APPL1, p-AMPK/T-AMPK, and PPAR*α* expression and LV +*dP/dt* (*P* < 0.05) compared with normal rats. Exenatide treatment significantly increased the adiponectin and HMW adiponectin concentration; the expression levels of APPL1, p-AMPK/T-AMPK, and PPAR*α*; and LV +*dP/dt* (*P* < 0.05), while the concentrations of TC and TG, the apoptosis rate, the LVEDP, and the expression level of NF-*κ*B were significantly decreased (*P* < 0.05) compared with the D and DI groups. These findings suggest that these effects were independent of glucose control. Thus, we conclude that exenatide could increase adiponectin and HMW adiponectin secretion and activate the APPL1-AMPK-PPAR*α* axis to inhibit myocardial cell apoptosis and improve cardiac function.

ER stress in diabetes plays a crucial role in diabetic cardiovascular complications and cardiomyocyte apoptosis. Glucose-regulated protein 78 (GRP78), also called immunoglobulin heavy chain binding protein (BIP), is a molecular chaperone of ER stress sensor PKR-like ER kinase (PERK), inositol-requiring enzyme-1A (IRE1A), and activating transcription factor-6 (ATF6) and is often used as a parameter to evaluate ER stress [24]. Our experiment also suggested that diabetic rats express significantly more GRP78 than normal rats (*P* < 0.05). After the exenatide intervention, the level of GRP78 expression was significantly decreased compared with the D and DI groups (*P* < 0.05). The GRP78 expression level is positively correlated with the apoptosis rate and the level of NF-*κ*B expression and is negatively correlated with the adiponectin and HMW adiponectin concentration, cardiac function, and the expression level of APPL1, AMPK, and PPAR*α*. Thus, we conclude that exenatide acts via the APPL1-AMPK-PPAR*α* axis to ameliorate cardiomyocyte apoptosis and improve diabetic cardiac function through improvement of ER stress.

AMPK is a key protein in metabolism control. As a serine/threonine protease that can be activated under conditions of energy deficiency, AMPK plays an important role in regulating the energy metabolism in cells. In addition to GLP-1, metformin and DPP-4 inhibitors are also AMPK inducers. Metformin is commonly applied in type 2 diabetes. In addition to reducing glucose levels and improving insulin resistance, metformin also can protect cells by activation AMP and inhibiting fatty acid oxidation and oxidative stress. AMPK activation by metformin is associated with improving diabetic and nondiabetic patients' cardiac function [25, 26]. Barreto-Torres et al. found that metformin induced AMPK and downstream PPAR*α* activation to protect H9C2 cells against oxidative stress [27]. In addition, some researchers hold that metformin can reduce plasma DPP-4 activity and increase circulating levels of GLP-1, suggesting that it may act as a DPP-4 inhibitor [18]. Many studies have suggested that DPP-4 inhibitors have cardioprotective effects, especially during ischemia-reperfusion (I/R) injury in both animal models and clinical studies [28]; these effects may be associated with AMPK, but the detailed mechanism is still unknown [29, 30]. The SAVOR-TIMI-53 and EXAMINE trials revealed that saxagliptin and alogliptin do not increase the rate of diabetes-related cardiovascular events [31, 32]. However, the SAVOR-TIMI-53 trial and another meta-analysis suggested that DPP-4 inhibitors could increase the rate of hospitalization for heart failure in patients with type 2 diabetes [33]. Therefore, it is unclear whether these results are independent effects of DPP-4 inhibitors or downstream GLP-1 effects. Whether these effects are involved in the APPL1-AMPK-PPAR*α* axis needs more evidence and further investigation.

Although GLP-1R is the main receptor for GLP-1 and its isoforms, including GLP-1(7-36) and GLP-1(9-36), in GLP-1R knockout mice, GLP-1(9-36) can reverse cardiac dysfunction. It has been suggested that there may be a GLP-1R-independent manner to improve cardiovascular function [34]. Further research has revealed that FAT/CD36 may be another potential receptor to separate GLP-1 and its isoforms into GLP-1(28-36) and (32-36), inhibiting fatty acid oxidation in mitochondria and ROS production, reducing the level of oxidative stress, and protecting cells from apoptosis [35]. Our research suggests that adiponectin and the APPL1-AMPK-PPAR*α* axis are important targets to interpret the cell protection mechanism of GLP-1, and this axis may be a potential target to prevent diabetic cardiovascular complications in the future. However, whether exenatide activates FAT/CD36 to reduce cardiomyocyte apoptosis needs further investigation.

Our research and conclusions were based on diabetic rats, and further research into the overexpression and inhibition of APPL1 in vivo and in vitro is needed to investigate the detailed mechanism of APPL1 activity. In addition to their role in ER stress, whether GLP-1 and the APPL1-AMPK-PPAR*α* axis (via mitochondrial dysfunction) participate in diabetic cardiomyocyte apoptosis and cardiac dysfunction is still unknown. These specific mechanisms still require further investigation and exploration.

## Figures and Tables

**Table 1:** Metabolic parameters of rats.

Groups	Body weight (g)	FBG (mmol/L)	Fins (mU/L)	TG (mmol/L)	TC (mmol/L)	Adiponectin (*µ*g/L)	HOMA-IR	HMW-adiponectin (*µ*g/L)
Control (*n* = 8)	318.1 ± 6.9^&^	4.96 ± 0.25^&^	21.85 ± 2.30	0.34 ± 0.04^&^	1.32 ± 0.04^&^	0.52 ± 0.06^&^	4.81 ± 0.48^&^	0.43 ± 0.11^&^
D (*n* = 8)	457.8 ± 25.0^*∗*^	7.43 ± 0.83^*∗*^	21.37 ± 1.60	0.78 ± 0.06^*∗*^	2.16 ± 0.07^*∗*^	0.47 ± 0.04^*∗*^	7.35 ± 0.74^*∗*^	0.34 ± 0.09^*∗*^
DI (*n* = 8)	450.6 ± 6.0^*∗*^	5.23 ± 0.34	25.66 ± 1.88^*∗*&^	0.75 ± 0.07^*∗*^	2.15 ± 0.07^*∗*^	0.47 ± 0.03^*∗*^	5.81 ± 0.37^&^	0.38 ± 0.08
DE (*n* = 8)	318.7 ± 10.7^&^	5.10 ± 0.33	23.20 ± 1.47	0.52 ± 0.04^&^	1.30 ± 0.08^&^	0.76 ± 0.03^*∗*&^	5.27 ± 0.59^&^	0.70 ± 0.07^&*∗*^

FBG, fasting blood glucose; FFA, free fatty acids; FIns, fasting plasma insulin; kg twice daily; Control, rats fed normal chow diet; TC, total cholesterol; TG, triglyceride. Data are means ± s.e.

Compared with the Control group: ^*∗*^ *P* < 0.05; compared with the D group: ^&^ *P* < 0.05.

**Table 2:** The results of cardiac function testing.

	LVSP (mmHg)	+*dP*/*dt* (mmHg/s)	−*dP*/*dt* (mmHg/s)	LVEDP (mmHg)
Control	126.25 ± 4.59^*∗*#^	604.55 ± 6.59^*∗*#^	623.48 ± 8.85^*∗*#^	10.38 ± 0.93^*∗*#^
D	105.87 ± 4.08	513.86 ± 10.54	430.48 ± 27.37	17.62 ± 1.74
DI	107.19 ± 4.09	519.31 ± 12.61	417.64 ± 18.67	17.48 ± 1.49
DE	119.11 ± 5.11^*∗*#^	580.43 ± 14.72^*∗*#^	548.76 ± 24.40^*∗*#^	13.64 ± 1.25^*∗*#^

^*∗*^Compared with the D group, *P* < 0.05.

^#^Compared with the DI group, *P* < 0.05.

**Table 3:** The apoptosis rate of rats' myocardial cells.

	Control	D	DI	DE
Apoptosis rate	6.14% ± 2.32%^**∗**#^	55.71% ± 3.84%	43.91% ± 4.23%	27.43% ± 3.63%^**∗**#^

^*∗*^Compared with the D group, *P* < 0.05.

^#^Compared with the DI group, *P* < 0.05.

**Table 4:** Protein expression levels of APPL1, p-AMPK/T-AMPK, PPAR*α*, GRP78, and NF-*κ*B.

	Control	D	DI	DE
APPL1	0.75 ± 0.03^**∗**#^	0.36 ± 0.03	0.36 ± 0.04	0.65 ± 0.02^**∗**#^
p-AMPK/	0.94 ± 0.02^**∗**#^	0.58 ± 0.04	0.60 ± 0.03	0.78 ± 0.04^**∗**#^
T-AMPK	1.14 ± 0.13	1.06 ± 0.03	1.12 ± 0.12	1.05 ± 0.09
p-AMPK/T-AMPK	0.83 ± 0.08^**∗**#^	0.55 ± 0.03	0.55 ± 0.09	0.75 ± 0.04^**∗**#^
PPAR*α*	1.89 ± 0.03^**∗**#^	1.25 ± 0.15	1.27 ± 0.05	1.72 ± 0.05^**∗**#^
GRP78	0.44 ± 0.04^**∗**#^	0.77 ± 0.05	0.70 ± 0.05	0.34 ± 0.02^**∗**#^
NF-*κ*B	0.35 ± 0.05^**∗**#^	1.39 ± 0.08	1.41 ± 0.08	0.58 ± 0.09^**∗**#^

^*∗*^Compared with the D group, *P* < 0.05; ^#^Compared with the DI group, *P* < 0.05.
